# Characterizing Advanced Parkinson's Disease: Romanian Subanalysis from the OBSERVE-PD Study

**DOI:** 10.1155/2021/6635618

**Published:** 2021-01-25

**Authors:** Jozsef Attila Szasz, Dragos Catalin Jianu, Mihaela Adriana Simu, Viorelia Adelina Constantin, Adriana Octaviana Dulamea, Koray Onuk, Diana Popescu, Mihai-Titus Vasile, Bogdan Ovidiu Popescu, Alfonso Fasano, Ovidiu Alexandru Bajenaru

**Affiliations:** ^1^Department of Neurology, Mureș Emergency Clinical County Hospital, “George Emil Palade” University of Medicine, Pharmacy, Science and Technology, Târgu Mureș 540139, Romania; ^2^Department of Neurology, “Victor Babes” University of Medicine and Pharmacy, Timisoara 300041, Romania; ^3^Fundeni Clinical Institute, “Carol Davila” University of Medicine and Pharmacy, Bucharest 020021, Romania; ^4^AbbVie Inc., North Chicago, Chicago, IL 60064, USA; ^5^AbbVie SLR, Bucharest 020276, Romania; ^6^Department of Neurology, University Emergency Hospital, “Carol Davila” University of Medicine and Pharmacy, Bucharest 020021, Romania; ^7^Department of Neurology, Colentina Clinical Hospital, “Carol Davila” University of Medicine and Pharmacy, Bucharest 020021, Romania; ^8^Edmond J. Safra Program in Parkinson's Disease and Morton and Gloria Shulman Movement Disorders Clinic, Toronto Western Hospital and Division of Neurology, UHN, Division of Neurology, University of Toronto, Toronto, ON, Canada 7MCL-402; ^9^Krembil Research Institute, Toronto, ON, Canada M5T0S8

## Abstract

OBSERVE-PD was a cross-sectional, multicountry, observational study conducted in 128 Movement Disorders Centers (MDCs) in 18 countries. Overall, the study enrolled 2615 patients. The aim was to determine the proportion of patients with advanced Parkinson's disease (APD) versus non-APD from MDCs and to uncover the clinical burden of APD, as well as a correlation between overall assessment of APD and several indicators of APD. The advanced stage of the disease and severity were assessed by investigators using their clinical judgement. Data were collected during a single visit between February 2015 and January 2016. Agreement on physician judgement of APD diagnosis and fulfillment of at least one previously established APD indicator was calculated. Motor and nonmotor symptoms (NMSs), activities of daily living, treatment complications, quality of life (QoL), conventional treatments, and device-aided therapy (DAT) eligibility were assessed. Here, country-specific results of 161 Romanian patients with PD are presented. In total, 59.0% of patients were diagnosed with APD and 78.8% met at least one APD indicator. There was only moderate agreement between clinical judgement of APD and overall fulfillment of APD indicators. All scores related to motor symptoms, NMSs, and treatment complications, as well as to QoL, showed a higher disease burden for patients with APD versus non-APD. Physicians considered 73.7% of patients with APD eligible for DAT. The majority of patients eligible for DAT (54.3%) did not receive such treatment. Our results highlight the importance of earlier recognition of APD, by combining clinical judgement with more standardized clinical tools, such as generally recognized APD criteria. However, timely diagnosis of APD alone is not enough to improve patient outcomes. Other critical factors include patient acceptance and access to appropriate treatment.

## 1. Introduction

Parkinson's disease (PD) is a common chronic, progressive, neurodegenerative disease. Over the course of the disease, PD may lead to severe disability despite optimal treatment [1, 2]. The prevalence of PD was estimated at 1% of the population over 60 years [3] or 6.1 million persons worldwide. The Romanian PD population is estimated at 40,517 patients according to the Global Burden of Disease study [4].

The etiology of PD lies in the degeneration of dopamine-producing cells in substantia nigra [5]. The resulting lack of dopamine is contributory to the clinical cardinal signs of PD: bradykinesia plus resting tremor or muscular rigidity [6].

The most effective therapy for many patients is levodopa, still the gold standard for this disease [7]. Unfortunately, treatment becomes less effective over the years because many patients experience an increased sensitivity to subtle fluctuations of levodopa plasma levels, which ultimately narrows the therapeutic window of the drug. In consequence, levodopa-related complications, such as motor fluctuations or dyskinesia, may occur [8–10]. Identification of advancing Parkinson's disease is particularly relevant since it is linked to a marked decrease in health-related quality of life (QoL) [11–13].

To date, no consensus on how to specifically define each PD stage is in place, and identification of “advanced”/“complex” PD is still a matter of debate [14, 15]. However, physicians often prefer the clinical evaluation and prior history to determine staging in PD because the Hoehn and Yahr (H&Y) scale does not capture motor fluctuations, dyskinesia, or nonmotor symptoms (NMSs) that are typically seen in patients with PD in the advanced stage [16]. Generally, PD is considered “advanced” when the patient experiences a fluctuating clinical state with unpredictable alternating periods of good symptom control (“on” stage) and poor symptom control (“off” stage), despite adequate conventional therapy [17–19]. These fluctuations may affect both motor symptoms and nonmotor symptoms [17]. Some previous studies indicated that motor complications develop in 10% of patients with PD per year, marking the transition to advanced PD (APD) [20, 21]. Device-aided therapy (DAT), including continuous infusion therapies such as subcutaneous apomorphine and intraduodenal levodopa, and stereotactic surgery are considered in patients with APD uncontrolled after multiple attempts to optimize the conventional therapy [21–23].

In Romania, PD diagnosis and management is led by general neurologists. General practitioners (GPs) or other specialty physicians (such as gastroenterologists, urologists, rheumatologists, and internal medicine specialists) refer neurological patients with suspicion of PD to general neurologists from outpatient clinics. They certify the PD diagnosis and initiate the antiparkinsonian conventional treatment, following the Parkinson Disease Diagnosis and Treatment Guidelines [24] of the Romanian Society of Neurology and recommendations from international expert groups [18, 25–27]. For a second opinion on diagnosis and for treatment optimization, outpatient clinic general neurologists may also refer patients with PD to university clinics acting as MDCs with higher expertise in PD. However, the APD diagnosis may be established long after these patients have transitioned to the advanced stage.

Romanian experience with levodopa/carbidopa intestinal gel (LCIG) and deep brain stimulation (DBS) is more than 11 years. Recent publications present details on Romanian expertise [28–31] regarding DAT-eligible patient profiles and LCIG treatment. Our clinical practice follows the current international recommendations [21, 26, 32–34] of involving a dedicated multidisciplinary team (neurologist, gastroenterologist, psychiatrist, anesthesiologist, and nurse with expertise in PD and DATs) and an aftercare system. Testing and titration of LCIG are performed during hospitalization. For all eligible patients selected for LCIG, we propose therapy testing by nasojejunal administration [24].

To better characterize and understand APD in various real-world settings, an observational, cross-sectional study (OBSERVE-PD) was conducted in 128 Movement Disorder Centers (MDCs) from 18 countries. The purpose of this study was to evaluate the proportion of patients with APD versus non-APD presenting in MDCs and to compare demographics, treatment regimens, disease status, and quality of life (QoL) between these two groups of patients. Additionally, this study evaluated the level of agreement between physician judgement of an APD diagnosis, APD indicators fulfillment, and DAT-eligibility characteristics [35].

Here we present these results for patients enrolled in Romania. In addition, we performed a supplementary analysis by APD indicator fulfillment to better characterize the APD/non-APD groups, as well as DAT-eligibility parameters.

## 2. Materials and Methods

OBSERVE-PD was a cross-sectional, observational, multinational, multicenter study conducted in 18 countries between February 2015 and January 2016. The study design was previously reported [35].

### 2.1. Study Objectives

The primary objective was to evaluate the proportion of patients identified with APD according to the clinical judgement of neurologists with expertise in movement disorders from participating MDCs. The secondary objectives included a comparison of the APD diagnosis assessed by physician clinical judgement and proposed APD indicator fulfillment, respectively. Predefined objectives also included the characterization of clinical and nonclinical features of the two subgroups (APD and non-APD). Motor and nonmotor symptoms, treatment complications, activities of daily living (ADL), and quality-of-life end points were assessed.

### 2.2. Study Setting

From Romania, 15 MDCs (neurology departments at university hospitals with experience in movement disorders) participated in OBSERVE-PD and enrolled patients. The study investigators were neurologists (further noted as physicians) with expertise in movement disorders from the participating MDCs.

### 2.3. Inclusion and Exclusion Criteria

Patients diagnosed with PD attending a routine clinical visit at an MDC were asked to participate in the study. Enrolled patients had to be at least 18 years of age. To avoid bias regarding disease management in normal clinical practice, patients were not included in the study if they were in the “off” stage during their study visit, if the diagnosis of PD was not certain, or if patients were participating in a concurrent clinical study [35].

For each patient, data of one single visit were collected as far as they were part of the normal routine. All patients signed an informed consent form. In all participating countries, the study was approved by local ethics committees. The stipulations of the Declaration of Helsinki were adhered to.

### 2.4. Study Records and Assessments

Data on demographics (age, sex, residence, and caregiver support), cognitive status (general evaluation as in clinical practice), and comorbidities were gathered. History of PD (duration of disease, motor fluctuations, referral history, and disease stage based on physician judgement [advanced vs. nonadvanced]) and data on previous and current treatment (type and number of current treatments and treatment response) were also collected. DAT eligibility was dictated by physician judgement.

For disease characterization, physicians were asked to complete several questionnaires. The four parts of the Unified Parkinson's Diseases Rating Scale (UPDRS) were used [36]. Motor symptoms assessed during the study visit included UPDRS in “on” stage (Part II [ADL], Part III [motor complications], and Part IV [items 32, 33, 34, and 39; complications of therapy]). In addition, the UPDRS Part V (modified H&Y scale) was used to assess PD motor symptoms, staging, and relative level of disability. NMSs over the last month were assessed with the Non-Motor Symptoms Scale for Parkinson's Disease (NMSS) [37]. To evaluate QoL, patients completed the 8-Item Parkinson's Disease Questionnaire (PDQ-8) [38].

Throughout the entire text, “APD” refers to the diagnosis established by physician judgement. To assess APD status, the physicians used first their clinical judgement and then evaluated fulfillment of each APD indicator, which were developed by the Delphi method, established by expert consensus, and validated by general neurologists [32, 39–44], as prespecified in the study protocol (Table 1) [35]. The symptom severity terms related to the Delphi criteria (e.g., mild, moderate, and severe) and description of the symptoms were defined by a consensus of international movement disorders experts, in Round 3 of a Delphi panel, as follows: mild: detectable to clinician but not interfering with daily life (minimally troublesome to the patient or not troublesome at all); moderate: detectable to clinician and influences daily life (troublesome to the patient); severe: detectable to clinician and significantly influences daily life (very troublesome to the patient) [32].

### 2.5. Statistical Analysis

All enrolled patients for whom physician judgement on PD stage was available were included in the statistical analysis. The SAS^®^ package version 9.2 was used for Cohen's Kappa statistic disease characterization (UPDRS, NMSS, and PDQ-8 scores). Statistical analyses were performed using the *R* package version 4.0.0 for all other comparisons between groups.

Descriptive statistics were conducted for categorical, ordinal, and ratio variables for all patients, as well as for patients with APD and non-APD separately. Proportions, quartiles, means, confidence intervals for the means, and standard deviations were reported.

Student's *t*-test was used to test for differences in the means of ratio variables by patient category. The *t* statistic, the degrees of freedom, and the *P* value were reported. The size of the effect was computed when *P* ≤ 0.05. The Mann–Whitney–Wilcoxon test was used to test for differences in the distribution of ordinal variables by patient category. The mean ranks for each group and the *P* value were reported. The *χ*^2^ and Fisher's exact tests were used to test for differences in the distribution of categorical variables by patient category. *χ*^2^ and the degrees of freedom were reported for the former, as well as the *P* value for both tests. One-proportion *Z*-test for one sample was used to test for differences between proportions in the distribution of dichotomous variables. *χ*^2^, the degrees of freedom, and the *P* value were reported. Cohen's Kappa statistic [45] was calculated to measure agreement between physician judgement of APD diagnosis and each of the eleven APD indicators and the cumulative APD indicators score. Higher Kappa values indicate better agreement (≤0.20, no/slight agreement; 0.21–0.40, fair agreement; 0.41–0.6, moderate agreement; 0.61–0.80, substantial agreement, and 0.81–1.00, almost perfect agreement) [45].

## 3. Results

### 3.1. Overall Patients and Disease Characteristics

In total, 161 patients fulfilled the inclusion criteria and were included in the analysis. According to physician judgement, 95 patients (59.0%) were diagnosed with APD and 66 (41.0%) with non-APD. Demographic and clinical characteristics, such as age, sex, and presence of comorbidities, were not statistically different between APD and non-APD groups (Table 2). Significantly more patients with APD compared with non-APD had 3 and 4 H&Y stage and required more caregiver support (*P* < 0.0001 for all). Patients with APD also had a higher mean duration since PD diagnosis, more motor fluctuations (*P* < 0.0001 for all), and worse cognitive dysfunction (*P* < 0.001) (Table 2). Although significantly more patients with APD versus non-APD had motor fluctuations, the mean duration of motor fluctuations expressed in years was similar in both groups. Most patients (APD: 91.6%; non-APD: 93.9%) had one or more comorbidities, without a statistical difference between the two groups (Supplementary Table S1).

### 3.2. Patient Referral to MDCs

In patients referred to the participating MDCs, the mean time since referral was 2.2 and 1.4 years in patients with and without APD, respectively. Significantly more patients were referred to MDC by a neurologist (50.0% vs. 11.1%) in the APD group and by a general practitioner (GP) (63.0% vs. 37.0%) in the non-APD group (Table 3). The main reasons for APD patients' referral as documented in the referral note included in their medical files were “PD progression” to APD and development of persistent motor complications and “to consider DAT initiation.” The main reasons for referral for patients with non-APD were to reconfirm diagnosis and reevaluate treatment for obtaining symptom control.

### 3.3. Conventional Treatments

Patients with APD versus with non-APD received a significantly higher mean number of conventional treatments (2.58 vs. 1.95, respectively; *P* < 0.001) (Table 4). The most frequent therapy was oral levodopa associated with carbidopa or benserazide (APD: 84.2%; non-APD: 75.8%), followed by monoamine-oxidase B (MOAB) inhibitors (APD: 55.8%; non-APD: 43.9%) and oral dopamine agonists (APD: 37.9%; non-APD: 51.5%).

### 3.4. PD Rating Scale Scores

All mean UPDRS and total NMSS scores were significantly higher (*P* < 0.0001) in patients with APD than with non-APD (Supplementary Table S2). The mean UPDRS II scores for ADL were 20.6 and 9.3 in patients with APD and with non-APD, respectively. The mean UPDRS IV scores for duration of dyskinesia were on average 1.1 and 0.3 in patients with APD and with non-APD, respectively. Dyskinesia and dyskinesia-related disability and pain were reported by more patients with APD (70.2%, 58.5%, and 39.4%, respectively) than with non-APD (18.5%, 14.3%, and 4.8%, respectively). Most patients with APD (90.5%) reported “off” time at least 25% of the day.

The NMSS score was not documented in many patients and therefore was evaluated only in a subgroup of patients (*n* *=* 108). Significantly more patients with APD (73.6%) than with non-APD (36.4%) had severe or very severe NMSs (*P* < 0.001). Impairment of QoL was significantly higher in patients with APD (mean PDQ-8: 51.8) compared with non-APD (mean PDQ-8: 29.2) (*P* < 0.0001).

### 3.5. APD Diagnosis Agreement: Physician Judgement and APD Indicators

Physicians established APD and non-APD diagnoses by clinical judgement. Reflecting the agreement between APD diagnosis and fulfillment of APD indicators, 94.7% of patients with APD also met at least one APD indicator and 43.9% of patients with non-APD did not meet any APD indicator (Table 5). Regarding lack of agreement, 5.3% of patients with APD did not meet any APD indicator and 56.1% of patients with non-APD met at least one APD indicator.

In general, a moderate agreement between physician judgement and overall fulfillment of APD indicators was observed. The highest agreement between physician judgement and meeting of at least one APD indicator was achieved for limitations in ADL (Cohen's Kappa: 0.593), followed by NMSs fluctuations (Cohen's Kappa: 0.571) and troublesome motor fluctuations (Cohen's Kappa: 0.555) (Table 6).

### 3.6. Characterization of APD Study Population by APD Indicators

Significantly more patients with APD had moderate/severe motor and NMSs fluctuations (*P* < 0.01), as well as moderate/severe limitation of ADL (*P* < 0.0001) (Table 7). In contrast, patients with APD had significantly fewer falls and less moderate/severe dementia or psychosis (*P* < 0.0001).

### 3.7. DAT Eligibility: Patients and Disease Characteristics

Overall, physicians considered 73.7% of patients with APD eligible for DAT. For 45.5% of these patients, DAT was ongoing or about to start. Of all patients with APD, the mean time since diagnosis was significantly higher in DAT-eligible versus DAT-ineligible groups (10.7 years vs. 6.2 years, *P* < 0.001). Significantly more patients with APD requiring caregiver support were considered DAT-eligible versus DAT-ineligible (*P* < 0.01). The mean time since diagnosis and the mean number of years with motor fluctuations were significantly higher (*P* < 0.001) in DAT-eligible versus DAT-ineligible patients (Supplementary Table S3). Similarly, H&Y stage was higher in DAT-eligible versus DAT-ineligible patients (*P*=0.023). Although there was no difference regarding the presence of motor fluctuations at the moment of study visit, APD DAT-eligible patients presented longer-term motor fluctuations than DAT-ineligible (4.1 years vs. 2.2 years, *P* < 0.001). In the overall patient population diagnosed with APD, significantly more DAT-eligible versus DAT-ineligible patients (*P*=0.037) presented troublesome dyskinesia ≥2 hours of the day (Table 8). The most frequent reasons in eligible patients with APD not receiving or planning to receive DAT were patient-related: either needing more time to decide (60.5%) or patient refusal (18.4%) (Table 9).

## 4. Discussion

OBSERVE-PD, a multicountry, observational, cross-sectional study, collected information on patients with PD who were admitted to specialized MDCs. The study was conducted in Canada, Australia, 15 European countries, and Israel and included in total 2615 patients [35]. All participants have been referred to specialized MDCs for further evaluation or treatment. The data presented here focus on the subset of 161 Romanian patients from 15 MDCs.

### 4.1. Overall Patients and Disease Characteristics

The OBSERVE-PD study captured a snapshot of clinical practice in different MDCs in international setting. We noticed a significantly higher caregiver support requirement for patients with APD versus non-APD. The result is not unexpected considering that increasing disability defines more advanced stages of the disease [16]. Although more patients with APD (92.6%) had motor fluctuations at the study visit versus patients with non-APD (7.4%), the mean number of years with this symptom is similar in the two groups (3.6 and 2.9 years, respectively).

When determining the transition to APD and potential treatment changes, according to the current guidelines and recommendations [18, 24–27, 32, 33], physicians should consider first some particular traits of motor fluctuations, such as severity, related disability, lack of response to treatment, and the dynamics rather the number of years of motor fluctuations duration.

The PD population enrolled in OBSERVE-PD showed a certain extent of homogeneity (e.g., mean age, mean number of years with motor fluctuations, mild cognitive dysfunction, and distribution in 2.5–4 H&Y stages).

The proportion of patients with any comorbidities was somewhat higher in Romania (APD: 91.6%, non-APD: 93.6%) compared with the global OBSERVE-PD study population (APD: 89.1%, non-APD: 84.0%). Cardiac abnormalities and cardiovascular disease were more frequent in patients with APD from Romania (30.5%) than in the global study population (20.8%), which is not surprising since Romania has the highest incidence of cardiovascular death in Europe, and cardiovascular diseases are an important burden in the general population [46, 47]. In contrast, cognitive dysfunction was more frequent in patients in the global study (APD: 53.9%, non-APD: 35.2%) than in the Romanian substudy (APD: 46.3%, non-APD: 19.7%).

### 4.2. Conventional Treatments and Referral to MDCs

Despite expert recommendation [21, 33], in Romania some patients with PD are not referred to MDCs in a timely manner, even if motor fluctuations have occurred. As shown by our clinical practice and international expert opinion, some general neurologists struggle with multiple dose adjustments and combinations of oral drugs for so long that they delay referral to an MDC, resulting in a loss of potential initiation of a DAT in eligible patients and less likelihood for the patient of reaching treatment optimization [21].

Duration of PD, referral history, and number and types of PD treatment did not differ essentially between the Romanian substudy and the global study. In our study, most patients with APD were referred by neurologists because PD progressed to APD and they developed persistent treatment complications and also to consider DAT initiation, whereas most patients with non-APD were referred to the MDC by the GPs and other specialties for symptom control and diagnostic purposes, although this would be the general neurologist competency. Referral to MDCs for patients with non-APD might use MDC resources otherwise needed for the management of patients with APD and place a supplementary burden on the healthcare system. This alternative route bypassing the general neurologist suggests an unmet need to improve collaboration between the GP and referring neurologist. In our study, the number and type of conventional treatments were not different between the APD and non-APD groups, considering the actual treatment recommendations for the respective disease stages [18, 24–27].

### 4.3. PD Rating Scale Scores

For Romanian patients, disease burden was higher in patients with APD versus non-APD, as shown by UPDRS, NMSS, and PDQ-8 scores. Impairment of QoL was stronger in Romanian patients (APD: 51.8; non-APD: 29.2) compared with the global patient population (APD: 36.6; non-APD: 20.7).

In this context, the differences between patients with APD and non-APD in NMSS total score and its domains are noteworthy. In many cases with APD, NMSs, as well as treatment response fluctuations, are perceived by the patients as the most bothersome PD symptoms, thus largely contributing to the overall disease burden [48]. In our APD population, the mean UPDRS scores indicated mild dyskinesia: approximately 25% of “on” time with dyskinesia (mean score 1.1), mild dyskinesia-related disability (mean score 1.1), as well as absence or slight dyskinesia-related pain (mean score 0.7) [36]. Overall results show that, in APD, disease burden seems to correlate most with motor fluctuations and NMSs rather than with dyskinesia. This observation might help physician judgement for tailoring PD patient management to address patients' most important complaints first.

### 4.4. APD Diagnosis and Relevant Early APD Indicators

Delay in APD diagnosis is a globally recognized challenge [19, 21, 32, 33, 49]. The Delphi method has been used in previous studies to achieve consensus on the most relevant clinical indicators for APD developed by movement disorders experts [32]. Fifteen clinically important APD indicators have been identified; therefore, motor symptoms, such as troublesome motor fluctuations (APD indicator no. 1) or “off” time duration (UPDRS IV), as well as NMSs fluctuations (APD indicator no. 5), were ranked by general neurologists as relevant indicators for suspected APD.

The OBSERVE-PD study analyzed these APD indicators in real-world clinical settings from Romania to help characterize the advancing PD. Our results show that a diagnosis of APD was concluded in 59% of cases, a slightly higher percentage than the overall study level result observed at the global study level (51.3%). However, the percentages of patients with APD varied considerably across countries (e.g., Ireland: 24%; Czech Republic: 82%) [35]. The variability of APD diagnosis may be caused by the differences in the selection of MDCs and healthcare standards in each country, including different criteria for APD diagnosis, as currently, there is no global consensus on the matter [19, 32].

In the Romanian substudy and the total study population alike, there was only a moderate agreement between physician judgement regarding APD diagnosis and fulfillment of APD indicators (Cohen's Kappa in Romania: 0.418; in total study: 0.441). Similar to our findings, Martinez-Martin et al. [15] observed a moderate agreement (Cohen's Kappa: 0.48) between physician judgement and the classification of APD according to another questionnaire developed using the Delphi method (*Cuestionario de Enfermedad de Parkinson Avanzada* (CDEPA questionnaire) [19].

Although moderate, the highest agreement score between physician judgement and APD indicators was achieved for “limitations in ADL,” “moderate or severe troublesome motor fluctuations,” and “presence of nonmotor fluctuations.” In our study, however, significantly more patients with APD than non-APD met at least one of these three APD indicators mentioned above. In our practice, clinical judgement of APD diagnosis is primarily based on ADL impairment and presence of motor and nonmotor fluctuations, which are very bothersome for patients and reduce their QoL, as shown in previous studies [48, 50]. A recently published comprehensive review showed that physicians tend to consider advanced disease in patients with PD with a marked decrease in QoL and a high level of associated disability [18]. Therefore, their assessment in clinical practice should be made with priority by the less experienced neurologists.

However, with a lower agreement, the additional four indicators (≥5 times oral levodopa dosing, falls most of/all of the time, moderate or severe dementia, and moderate or severe psychosis) have been met in significantly more patients with APD than with non-APD. They might also show a high indicative value for APD diagnosis, complementing clinical judgement. More than half of the patients considered by the physicians as non-APD (56.1%) fulfilled at least one of the APD indicators (excepting “falls most of/all of the time”; Table 6). For these patients considered to have non-APD, the presence of at least one of the 10 APD indicators above might mark the transition to an early APD, requiring a closer and more frequent evaluation, and avoiding years of unnecessary impaired QoL [18, 32, 33, 51].

### 4.5. DAT Eligibility and Patient Characteristics

There were 73.7% and 66% of patients considered eligible for DAT in the Romanian APD group and the overall APD study population, respectively. Similarly, a retrospective study in patients with APD hospitalized during 2011 and 2017 in a university clinic in Romania [30] aiming to explore the clinical characteristics indicative for DAT initiation found that only 44% of the patients were considered eligible for DAT based on clinical judgement and available recommendations [19, 32, 33]. A potential explanation for this Romanian higher DAT eligibility might be earlier APD patient referral to MDCs (for a second expert opinion on treatment reevaluation). Patients with APD considered eligible for a DAT were significantly older, required caregiver support in a higher percentage, had more years since diagnosis, and had more years with motor fluctuations. Almost all DAT-eligible patients were equally distributed in H&Y stages 3 and 4, showing early/timely judgement of DAT consideration among Romanian study investigators.

### 4.6. Disease Characteristics in DAT-Eligible Patients (by APD Indicators)

The Delphi consensus mentioned above [32] identified eight clinically important APD indicators for DAT eligibility, of which four (troublesome dyskinesia, ≥2 hours of “off” time per day, sleep disturbances during the night, and limitation of ADLs) were also included in our list of APD indicators. Of these four indicators, in our findings, the presence of troublesome dyskinesia ≥2 hours per day was significantly more frequent in DAT-eligible versus DAT-ineligible patients. This indicator might trigger the DAT-eligibility assessment. Although these indicators are helpful for the overall picture of DAT eligibility in patients with APD, assessment of validated response criteria [23] to actual treatment is also helpful but was not analyzed in our study.

### 4.7. DAT Undertreatment in Eligible Patients

In all Romanian patients, LCIG was the only type of ongoing DAT; in the global study population, LCIG was the current DAT only in 38.3% the patients. At the time of this OBSERVE-PD study, apomorphine pump was not available, and one MDC could offer stereotactic surgery. The percentage of DAT-undertreated patients (not initiating or planning DAT) from eligible patients with APD was higher in Romanian patients compared with the total OBSERVE-PD study population (54.3% vs. 37.7%). In our study, the top two reasons for DAT undertreatment of eligible patients (not initiating or planning DAT) are *patient need of more time to decide* and *patient refusal of advanced treatment*. These reasons seem related to patients' reluctance for a more invasive therapy than conventional treatment or might be the result of the communication between the medical professional on the one hand and patient and family on the other hand. Other frequent arguments for DAT undertreatment are the high prevalence of comorbidities and lack of caregiver/family support.

Acceptance of DAT by patients and their families depends on the optimal moment of DAT recommendation by the physician, which itself is determined by timely referral to an MDC [33, 51]. A patient's consent and the adequate treatment plan are also associated with communication with the neurologist [52, 53]. Several publications pointed to the importance of early, gradual, and tailored communication of the future need of invasive treatment, setting realistic expectations accordingly [33, 51]. Other authors highlight the importance of recognizing the patient's perspective on their burden of disability identifying individual factors contributing to impaired QoL and the importance of selection and tailoring treatment strategies to meet the patient's expectations [21, 54–56]. These results underscore the bumpy and sinuous journey for patients with PD, spanning several months or years from referral to MDC and APD diagnosis, to acceptance of an advanced treatment that might reduce symptoms and ensure a better QoL.

### 4.8. Strengths and Limitations

There is a limited number of observational studies that assessed the proportion of patients with APD and evaluated their disease characteristics and treatments [17, 22]. To our knowledge, OBSERVE-PD is the first real-world characterization of disease traits, advanced-stage diagnosis, proposed clinical indicator fulfillment, and DAT eligibility of a large cohort of patients with PD. This Romanian subanalysis is the first characterization of a real-world, nationwide DAT-eligible PD population presenting at MDCs in clinical practice. The results are correlated with updates from the literature and expert points of view. They also add to information from the retrospective analysis previously mentioned [30] and provide insights into the proportion and characteristics of patients with APD in MDCs. In addition, DAT undertreatment of many eligible patients with APD has become apparent, which should be further investigated.

Differences between the Romanian and the global cohort might be influenced by a variety of factors, including the selection of sites, country-specific treatment referral, patient management, treatment availability, and healthcare standards.

Patients in the “off” state at the study visit were not foreseen to be enrolled, which may have caused bias by potentially not including some of the most severely affected patients.

While more than 2500 patients were included in the OBSERVE-PD study [35], the Romanian substudy comprised 161 patients and is in many aspects in accordance with the results of the total study. Therefore, we assume that our findings reflect clinical and patient characteristics as well as treatment, disease burden, and QoL in patients with PD.

## 5. Conclusions

This substudy of the OBSERVE-PD study compared a wide range of patient and disease characteristics, treatment, and QoL in patients with PD managed in specialized centers in real-life conditions in Romania. A high percentage (56.1%) of the total patients with PD, although considered by their physicians as patients with non-APD, had fulfilled at least one of the listed APD indicators. Our opinion is that a comprehensive APD identification should consider all aspects evaluated by these indicators. The presence of these clinical indicators might then complement clinical judgement, allowing the identification of patients with a more subtle transition to early APD that might be referred earlier to MDCs to benefit from timely APD diagnosis and DAT treatment, if eligible.

Clinical judgement and APD consensus indicators [32] together might provide the best clinical approach for earlier APD diagnosis, to help recognize promptly and more reliably the progression to APD, as well as earlier, optimal treatment for appropriate patients as an individualized strategy for each PD patient.

## Figures and Tables

**Table 1:** APD indicators developed by Delphi method.

APD indicator	Measurement for fulfillment
(1) Troublesome motor fluctuations	Moderate or severe
(2) “Off” time	≥2 hours/waking day
(3) Nighttime sleep disturbances	Moderate or severe
(4) Troublesome dyskinesia	≥2 hours/waking day
(5) Nonmotor symptoms	Presence of fluctuations
(6) “Off” time	At least every 3 hours
(7) Levodopa dosing	≥5 times daily
(8) Activities of daily living limitation	Moderate or severe
(9) Falling	Most of/all of the time
(10) Dementia	Moderate or severe
(11) Psychosis	Moderate or severe

APD: advanced Parkinson's disease.

**Table 2:** Demographics and disease-related characteristics.

Variable	APD (*n* = 95)	Non-APD (*n* = 66)	*P*
Mean age, years (SD)	67.0 (8.3)	65.5 (10.0)	0.31
Males, *n* (%)	57 (60)	30 (45.5)	0.097
**Requirement of caregiver support (yes), *n* (%)**	**82 (86.3)**	**31 (47)**	**<0.0001**
**Mean time since diagnosis, years (SD)**	**9.5 (5.1)**	**3.7 (3.7)**	**<0.0001**
**Motor fluctuations (yes), *n* (%)**	**88 (92.6)**	**7 (7.4)**	**<0.0001**
Mean time with motor fluctuations, years (SD)	3.6 (2.9)	2.9 (3.1)	0.48
Comorbidities (yes), *n* (%)	87 (91.6)	62 (93.9)	0.76
**Cognitive dysfunction (yes), *n* (%)**	**44 (46.3)**	**13 (19.7)**	**<0.001**
Mild	33 (75)	11 (84.6)	
Moderate	8 (18.2)	2 (15.4)	
Severe	3 (6.8)	0	
**Hoehn andYahr stage, *n* (%)**			**<0.0001**
Stage 1	0 (0)	5 (7.6)	
Stage 1.5	0 (0)	8 (12.1)	
Stage 2	0 (0)	20 (12.1)	
Stage 2.5	4 (4.2)	22 (33.3)	
Stage 3	55 (57.9)	8 (12.1)	
Stage 4	33 (34.7)	3 (4.5)	
Stage 5	3 (3.2)	0 (0)	

APD: advanced Parkinson's disease; SD: standard deviation. Statistically significant *P* values (0.05) are presented in bold.

**Table 3:** Referral history to MDC. APD versus non-APD.

Variable	APD (*n* *=* 95)	Non-APD (*n* *=* 66)	*P*
**Specialty of referring physician to MDC, *n* (%)**	**54 (56.8)**	**54 (81.8)**	**<0.01**
**GP**	**20 (37.0)**	**34 (63.0)**	**0.012**
**Neurologist**	**27 (50.0)**	**6 (11.1)**	**<0.0001**
Other specialty	7 (13.0)	14 (25.9)	0.14
Mean time since referral, years (SD)	2.2 (3.0)	1.4 (2.2)	0.11
Reasons for referral, *n* (%)			
**PD progression**	**28 (51.9)**	**14 (25.9)**	**<0.01**
**To allow access to DAT initiation**	**12 (22.2)**	**0 (0)**	**<0.001**
**Symptoms no longer controlled**	**6 (11.1)**	**20 (37)**	**<0.01**
**For diagnostic purposes**	**8 (14.8)**	**20 (37)**	**0.015**
Other reasons	3 (5.6)	4 (7.4)	1

APD: advanced Parkinson's disease; DAT: device-aided therapy; GP: general practitioner; MDC: movement disorder center; and SD: standard deviation. Statistically significant *P* values (0.05) are presented in bold.

**Table 4:** Actual conventional treatments. APD versus non-APD.

Variable	APD (*n* *=* 95)	Non-APD (*n* *=* 66)	*P*
**Treatment type,** ^**a,b**^ ***n* (%)**			
Oral levodopa + carbidopa or benserazide	80 (84.2)	50 (75.8)	0.22
MAOB inhibitor	53 (55.8)	29 (43.9)	0.15
Oral dopamine agonist(s)	36 (37.9)	34 (51.5)	0.11
COMT inhibitor	27 (28.4)	0 (0)	**<0.0001**
Amantadine	27 (28.4)	4 (6.1)	**<0.001**
Other^c^ medication	22 (23.2)	12 (18.2)	0.56
**Mean number of standard treatments/patients**	**2.58**	**1.95**	**<0.001**
1 treatment, *n* (%)	14 (14.7)	14 (21.2)	
2 treatments, *n* (%)	28 (29.5)	29 (43.9)	
3 treatments, *n* (%)	24 (25.3)	19 (28.8)	
4 treatments, *n* (%)	17 (17.9)	0 (0)	
5 treatments, *n* (%)	7 (7.4)	0 (0)	
No treatment specified,^d^*n* (%)	5 (5.3)	4 (6.1)	

APD: advanced Parkinson's disease; COMT: catechol-*o*-methyltransferase; and MAOB: monoamine-oxidase B. ^a^No data available for 5 APD and 4 non-APD patients, respectively; ^b^multiple entries were possible; ^c^including dopamine agonist patch; and ^d^no treatment specified/missing information. Statistically significant *P* values (0.05) are presented in bold.

**Table 5:** Overall agreement between APD diagnosis and fulfillment of APD indicators.

		Fulfillment of APD indicators		Level of agreement	
Diagnosis	Number of patients (%)	Yes, ≥1 *n* (%)	No, any *n* (%)	Cohen's Kappa	[95% CI]
**APD**	**95 (59)**	**90 (94.7)**	5 (5.3)	**0.418**	**[0.284−0.552]**
Non-APD	66 (41)	**37 (56.1)**	29 (43.9)		

APD: advanced Parkinson's disease; CI: confidence interval. Statistically significant *P* values (0.05) are presented in bold.

**Table 6:** Level of agreement between clinical judgement of APD diagnosis and fulfillment of each APD indicator.

Diagnosis	Number of patients (%)	APD indicator	Fulfillment of each APD indicator		Level of agreement	
			Yes, *n* (%)	No, *n* (%)	Cohen's Kappa	[95% CI]
**APD**	**95 (59)**	**Moderate or severe troublesome motor fluctuations**	**62 (65.3)**	**33 (34.7)**	**0.555**	**[0.437−0.673]**
**Non-APD**	**66 (41)**		**4 (6.1)**	**62 (93.9)**		
APD	95 (59)	≥2 hours of the waking day with “off” symptoms	57 (60.0)	38 (40.0)	0.487	[0.366**−**0.608]
Non-APD	66 (41)		5 (7.6)	61 (92.4)		
APD	95 (59)	Moderate or severe nighttime sleep disturbances	51 (53.7)	44 (46.3)	0.304	[0.168**−**0.439]
Non-APD	66 (41)		14 (21.2)	52 (78.8)		
APD	95 (59)	≥2 hours of the day with troublesome dyskinesia	40 (42.1)	55 (57.9)	0.333	[0.222**−**0.444]
Non-APD	66 (41)		3 (4.6)	62 (95.4)		
**APD**	**95 (59)**	**Presence of nonmotor fluctuations**	**74 (77.9)**	**21 (22.1)**	**0.571**	**[0.444−0.698]**
**Non-APD**	**66 (41)**		**13 (19.7)**	**53 (80.3)**		
APD	95 (59)	Presence of “off” time at least every 3 hours	42 (44.2)	53 (55.8)	0.343	[0.228**−**0.457]
Non-APD	66 (41)		4 (6.1)	62 (93.9)		
APD	95 (59)	≥5 times daily oral levodopa dosing	36 (37.9)	59 (62.1)	0.257	[0.143**−**0.370]
Non-APD	66 (41)		6 (9.1)	60 (90.9)		
**APD**	**95 (59)**	**Moderate or severe limitation of ADL capacity**	**77 (81.1)**	**18 (18.9)**	**0.593**	**[0.467−0.719]**
**Non-APD**	**66 (41)**		**14 (21.2)**	**52 (78.8)**		
APD	95 (59)	With falls most/all the time	14 (14.7)	81 (85.3)	0.124	[0.059**−**0.189]
Non-APD	66 (41)		0 (0.0)	66 (100.0)		

ADL: activities of daily living; APD: advanced Parkinson's disease; and CI: confidence interval. Cohen's Kappa values > 0.5 are presented in bold.

**Table 7:** Presence of APD indicators in the APD patient population.

Delphi indicator	Yes, *n* (%)	No, *n* (%)	*P*
**Moderate or severe troublesome motor symptoms fluctuations**	**62 (65.3)**	**33 (34.7)**	**<0.01**
≥2 hours of the waking day with “off” symptoms	57 (60.0)	38 (40.0)	0.065
Moderate or severe night sleep disturbances	51 (53.7)	44 (46.3)	0.54
≥2 hours of the day with troublesome dyskinesia	40 (42.1)	55 (57.9)	0.15
**Presence of NMS fluctuations**	**74 (77.9)**	**21 (22.1)**	**<0.0001**
Presence of “off” time at least every 3 hours	42 (44.2)	53 (55.8)	0.3
**≥5 times oral levodopa dosing**	**36 (37.9)**	**59 (62.1)**	**0.024**
**Moderate or severe limitation of ADL capacity**	**77 (81.1)**	**18 (18.9)**	**<0.0001**
**Falls most of/all of the time**	**14 (14.7)**	**81 (85.3)**	**<0.0001**
**Moderate or severe dementia**	**12 (12.6)**	**83 (87.4)**	**<0.0001**
**Moderate or severe psychosis**	**6 (6.3)**	**89 (93.7)**	**<0.0001**

ADL: activities of daily living; APD: advanced Parkinson's disease; and NMSs: nonmotor symptoms. Statistically significant *P* values (0.05) are presented in bold.

**Table 8:** DAT eligibility and fulfillment of each APD indicator.

APD indicator	DAT eligible, *n* (%)	DAT ineligible, *n* (%)	*P*
Moderate or severe motor fluctuations	46 (65.7)	16 (64)	1
≥2 hours of the waking day with “off” symptoms	43 (61.4)	14 (56)	0.64
Moderate or severe night sleep disturbances	37 (52.9)	14 (56)	0.82
**≥2 hours of the day with troublesome dyskinesia**	**34 (48.6)**	**6 (24)**	**0.037**
Presence of NMSs fluctuations	56 (80)	18 (72)	0.41
Presence of “off” time at least every 3 hours	33 (47.1)	9 (36)	0.36
≥5 times oral levodopa dosing	27 (38.6)	9 (36)	1
Moderate or severe limitation of ADL capacity	56 (80)	21 (84)	0.77
Falls most of/all of the time	12 (17.1)	2 (8)	0.34
Moderate or severe dementia	8 (11.4)	4 (16)	0.73
Moderate or severe psychosis	4 (5.7)	2 (8)	0.65

ADL: activities of daily living; APD: advanced Parkinson's disease; DAT: device-aided therapy; and NMSs: nonmotor symptoms. Statistically significant *P* value (0.05) is presented in bold.

**Table 9:** Reasons for not planning DAT in eligible patients with APD.

Reason^a^	Patients, *n* (%) (*n* *=* 38)
Patient needs more time to decide	23 (60.5)
Patient refusal	7 (18.4)
Lack of caregiver/family support	4 (10.5)
Presence of comorbidities	4 (10.5)
Motor function-related issues	2 (5.3)
Age	1 (2.6)
Cognitive-related issues	1 (2.6)
Psychiatric-related issues	1 (2.6)
Other reasons	4 (10.5)

APD: advanced Parkinson's disease; DAT: device-aided therapy. ^a^Multiple entries for each patient were possible.

## Data Availability

The data used to support the findings are available from the corresponding author upon resonable request. Previously reported data (primary manuscript and overall study results) were used to support this study and are available at https://doi.org/10.1186/s12883-019-1276-8. The overall study results are cited in the text as reference [24].

## Supplementary Materials

Supplementary Materials Supplementary Materials contain three supplementary tables. *Table S1*. Comorbidities. *Table S2*. Disease status and characteristics. *Table S3*. DAT eligibility—patients and characteristics.
