# Supplementary material for: Characterizing Advanced Parkinson's Disease: Romanian Subanalysis from the OBSERVE-PD Study
Source: Parkinsons Dis. 2021 Jan 25;2021:6635618. doi: 10.1155/2021/6635618 (PMC7850828; doi:10.1155/2021/6635618)
Supplement: Supplementary Materials — contain three supplementary tables. Table S1. Comorbidities. Table S2. Disease status and characteristics. Table S3. DAT eligibility—patients and characteristics. [file 6635618.f1.zip › 6635618.f1/Suppl Table S1.docx]

Table S1: Comorbidities, APD versus non-APD.

| Comorbidity | APD, n (%)  (*n =* 95) | Non-APD, n (%)  (*n =* 66) | *P* |
| --- | --- | --- | --- |
| Orthostatic hypotension | 11 (11.6) | 4 (6.1) | 0.28 |
| Chronic gastrointestinal disease | 9 (9.5) | 13 (19.7) | 0.1 |
| Cardiovascular disease | 29 (30.5) | 16 (24.2) | 0.48 |
| (Poly)neuropathy | 14 (14.7) | 7 (10.6) | 0.49 |
| Chronic renal disease/renal insufficiency | 4 (4.2) | 1 (1.5) | 0.65 |
| Chronic liver disease/liver insufficiency | 5 (5.3) | 4 (6.1) | 1 |
| Skin disease | 3 (3.2) | 4 (6.1) | 0.45 |
| Sleep disorders | 18 (18.9) | 17 (25.8) | 0.33 |
| Fatigability | 21 (22.1) | 20 (30.3) | 0.27 |
| Arterial hypertension | 33 (34.7) | 20 (30.3) | 0.61 |
| Diabetes mellitus | 11 (11.6) | 11 (16.7) | 0.36 |
| Chronic pulmonary disease | 3 (3.2) | 0 (0) | 0.27 |
| Depression | 23 (24.2) | 25 (37.9) | 0.08 |
| Other comorbidity | 31 (32.6) | 23 (34.8) | 0.87 |

APD: advanced Parkinson’s disease.
