# Supplementary material for: Characterizing Advanced Parkinson's Disease: Romanian Subanalysis from the OBSERVE-PD Study
Source: Parkinsons Dis. 2021 Jan 25;2021:6635618. doi: 10.1155/2021/6635618 (PMC7850828; doi:10.1155/2021/6635618)
Supplement: Supplementary Materials — contain three supplementary tables. Table S1. Comorbidities. Table S2. Disease status and characteristics. Table S3. DAT eligibility—patients and characteristics. [file 6635618.f1.zip › 6635618.f1/Suppl Table S2.docx]

Table S2: Disease status and characteristics.

|  | APD | | Non-APD | |  |
| --- | --- | --- | --- | --- | --- |
| Scale/questionnaire | n | Mean score (SD) | n | Mean score (SD) | Difference [95% CI] |
| UPDRS II: Activities of daily living | 95 | 20.6 (8.7) | 66 | 9.3 (5.8) | 11.3 [8.9, 13.7] |
| UPDRS III: Motor examination | 95 | 38 (18.7) | 66 | 22 (11.8) | 16.0 [10.9, 21.2] |
| UPDRS IV: Dyskinesia (duration) | 94 | 1.1 (1.0) | 65 | 0.3 (0.6) | 0.8 [0.5, 1.1] |
| UPDRS IV: Dyskinesia (disability) | 94 | 1.1 (1.1) | 63 | 0.2 (0.6) | 0.9 [0.6, 1.2] |
| UPDRS IV: Dyskinesia (pain) | 94 | 0.7 (1.1) | 63 | 0.1 (0.3) | 0.7 [0.4, 0.9] |
| UPDRS IV: Duration of “off” time | 95 | 1.4 (0.9) | 58 | 0.4 (0.6) | 1.0 [0.8, 1.3] |
| UPDRS V: Modified H&Y staging | 95 | 3.4 (0.6) | 66 | 2.2 (0.7) | 1.2 [1.0, 1.3] |
| Total NMSS score | 53 | 80.1 (53.8) | 55 | 33.8 (23.7) | 46.3 [30.6, 62.1] |
| Cardiovascular, including falls | 56 | 4.8 (5.0) | 60 | 1.6 (2.7) | 3.15 [1.67, 4.63] |
| Sleep/fatigue | 85 | 15.6 (10.6) | 61 | 5.4 (6.1) | 10.19 [7.21, 13.18] |
| Mood/cognition | 71 | 18.8 (15.7) | 61 | 6.9 (9.4) | 11.94 [7.39, 16.48] |
| Perceptual problems/hallucinations | 59 | 2.9 (5.5) | 60 | 0.9 (3.4) | 1.98 [0.34, 3, 63] |
| Attention/memory | 81 | 9.9 (9.4) | 59 | 3.9 (4.4) | 5.99 [3.38, 8.59] |
| Gastrointestinal | 79 | 9.1 (7.7) | 62 | 3.8 (4.4) | 5.30 [3.13, 7.47] |
| Urinary | 76 | 12.5 (9.2) | 64 | 5.7 (6.8) | 6.81 [4.07, 9,54] |
| Sexual function | 74 | 8.9 (8.5) | 60 | 6.1 (8.0) | 2.88 [0.04, 5.72] |
| Miscellaneous | 59 | 9.5 (7.5) | 59 | 4.7 (5.3) | 4.85 [2.47, 7.23] |
| PDQ-8 | 93 | 51.8 (21.8) | 65 | 29.2 (19.1) | 22.6 [16.0, 29.2] |

APD: advanced Parkinson’s disease; CI: confidence interval; H&Y: Hoehn & Yahr; NMSS: Non-Motor Symptoms Scale for Parkinson’s Disease; PDQ-8: 8-item Parkinson’s Disease Questionnaire; SD: standard deviation; UPDRS: Unified Parkinson’s Diseases Rating Scale.

*P* < 0.0001 for all mean score comparisons between patients with APD and non-APD, except for NMS sub-domain *perceptual problems/hallucinations* and *sexual function* where *P* = 0.0187 and 0.0468, respectively, for the difference in APD versus non-APD.
