# Supplementary material for: Characterizing Advanced Parkinson's Disease: Romanian Subanalysis from the OBSERVE-PD Study
Source: Parkinsons Dis. 2021 Jan 25;2021:6635618. doi: 10.1155/2021/6635618 (PMC7850828; doi:10.1155/2021/6635618)
Supplement: Supplementary Materials — contain three supplementary tables. Table S1. Comorbidities. Table S2. Disease status and characteristics. Table S3. DAT eligibility—patients and characteristics. [file 6635618.f1.zip › 6635618.f1/Suppl Table S3.docx]

Table S3: DAT eligibility⸻patients and disease characteristics.

| Variable | DAT eligible  (*n =* 70) | DAT ineligible  (*n =* 25) | *P* |
| --- | --- | --- | --- |
| Mean age, years (SD) | 65.4 (8.1) | 71.3 (7.6) | <0.01 |
| Males, n (%) | 46 (65.7) | 11 (44) | 0.096 |
| Requirement of caregiver support (Yes), n (%) | 65 (92.9) | 17 (68) | <0.01 |
| Mean time since diagnosis, years (SD) | 10.7 (5.2) | 6.2 (3.4) | <0.001 |
| Motor fluctuations (Yes), n (%) | 65 (92.9) | 23 (92) | 1 |
| Mean time with motor fluctuations, years (SD) | 4.1 (3.2) | 2.2 (1.2) | <0.001 |
| Presence of comorbidities (Yes), n (%) | 64 (91.4) | 23 (92) | 1 |
| Cognitive dysfunction (Yes), n (%) | 32 (45.7) | 12 (48) | 1 |
| Mild | 25 (78.1) | 8 (66.7) |  |
| Moderate | 5 (15.6) | 3 (25) |  |
| Severe | 2 (6.2) | 1 (8.3) |  |
| Hoehn & Yahr stage, n (%) |  |  | 0.023 |
| Stage 1 | 0 (0) | 0 (0) |  |
| Stage 1.5 | 0 (0) | 0 (0) |  |
| Stage 2 | 0 (0) | 0 (0) |  |
| Stage 2.5 | 3 (4.3) | 1 (4) |  |
| Stage 3 | 35 (50) | 20 (80) |  |
| Stage 4 | 30 (42.9) | 3 (12) |  |
| Stage 5 | 2 (2.9) | 1 (4) |  |

DAT: device-aided therapy; SD: standard deviation.
